# Leveraging large scale deep learning models for diagnosis and visual outcome prediction in retinitis pigmentosa

**DOI:** 10.1038/s41746-025-02311-9

**Published:** 2026-01-08

**Authors:** Tatsuya Nagai, Koya Homma, Yuto Kawamata, Masahito Yoshihara, Eiryo Kawakami, Takayuki Baba

**Affiliations:** 1 Department of Ophthalmology and Visual Science, Graduate School of Medicine, Chiba University, Chiba, Japan; 2 Department of Artificial Intelligence Medicine, Graduate School of Medicine, Chiba University, Chiba, Japan; 3 Institute for Advanced Academic Research (IAAR), Chiba University, Chiba, Japan; 4 Advanced Data Science Project (ADSP), RIKEN Information R&D and Strategy Headquarters, RIKEN, Yokohama, Kanagawa Japan

**Keywords:** Retina, Imaging and sensing

## Abstract

Retinitis pigmentosa (RP) is an inherited progressive retinal degeneration that shows symptoms of night blindness, visual field loss, declining of vision and eventually, blindness. Currently, gene therapy and retinal prosthesis are available, but the indication for these treatments is limited. In this study, we report on the development of a diagnostic and prognostic model for RP based on large-scale deep learning (DL) models pre-trained with fundus images. The EfficientNetB4 model performed best in diagnosing RP with an AUC of 0.94. The diagnosis of RP with this model is superior in cases with good vision. For visual prognosis, we applied machine learning survival analysis to DL-derived image features and clinical metadata, using a strict patient-level split to avoid data leakage. The hybrid model combining imaging and clinical data outperformed models based on either modality alone, especially in female patients. Time-dependent AUC analysis showed that prognostic performance was highest between 500 and 1400 days after examination. SHAP-based interpretability analysis revealed that the features contributing to RP diagnosis and those associated with prognosis were distinct. While our findings demonstrate the added value of fundus images in visual outcome prediction, further validation using external and multi-center datasets is necessary for clinical translation.

## Introduction

Retinitis pigmentosa (RP) is a well-known progressive genetic retinal dystrophy, with an estimated prevalence of 1 in 4000^1^. Cardinal symptoms of RP include night blindness, visual field constriction, and eventual decline in visual acuity and blindness, which significantly reduces the quality of life of patients. The second leading cause of legal blindness in Japan is RP, affecting about 20,000–30,000 patients. This progressive disease has a serious impact on the quality of life of patients.

Although there have been recent advances in the treatment of RP including gene therapy, such as voretigene neparvovec^2^ and retinal prosthesis including ARGUS II^3^, these therapies are quite costly and only available for a small part of patients. Therefore, no effective treatment has been generally established for this disease. Currently, the main practice is follow-up to monitor residual visual function and low vision care, in addition to the treatment of complications, such as cataracts or macular edema. Even after successful cataract surgery and mitigating the macular edema, the recovery of vision is limited by the remaining retinal function^4,5^. For patients with RP, it is important to plan their life stage and to schedule the use of social resources according to the visual impairment^6^. To conjecture visual prognosis is not easy because it differs from patient to patient, so it is desirable to develop methods for estimating visual prognosis.

In recent years, artificial intelligence (AI) based on deep learning (DL) has been utilized in various medical fields and its usefulness has been recognized firmly. Among the various fields in medicine, ophthalmology has a high affinity with AI because a large number of images are used for diagnosis, planning of treatment, and follow-up^7,8^. AI diagnostic models for various ophthalmic diseases, such as diabetic retinopathy and age-related macular degeneration have been reported^9–11^, and some of them have been put into practical use. Regarding RP, the diagnostic model using AI based on the ultrawide-field pseudocolor and autofluorescence fundus images has been reported^12^. The RP cases were successfully distinguished from normal controls with high sensitivity and specificity. Models to diagnose RP from fundus photographs or predict causative genes in other inherited retinal disorders including RP from fundus photographs and autofluorescence images have also been published^13^. The accuracy of RP diagnostic models is as high as 96%, showing high performance of the DL model regarding an assessment of RP in a cross-sectional manner^14^. However, there are few reports associated with AI-based prediction models of longitudinal visual functions or visual prognosis of RP^15,16^ because of the difficulty of collecting sufficient images for training the DL model and a lack of the complete set of vision for consecutive years combining images.

The purpose of this study is to develop a prediction model for visual acuity prognosis in patients with RP based on existing AI models using fundus photographs. In addition, we extract and analyze image features that affect the visual function and visual prognosis of RP.

## Results

### Characteristics of the study population

**Fig. 1:**
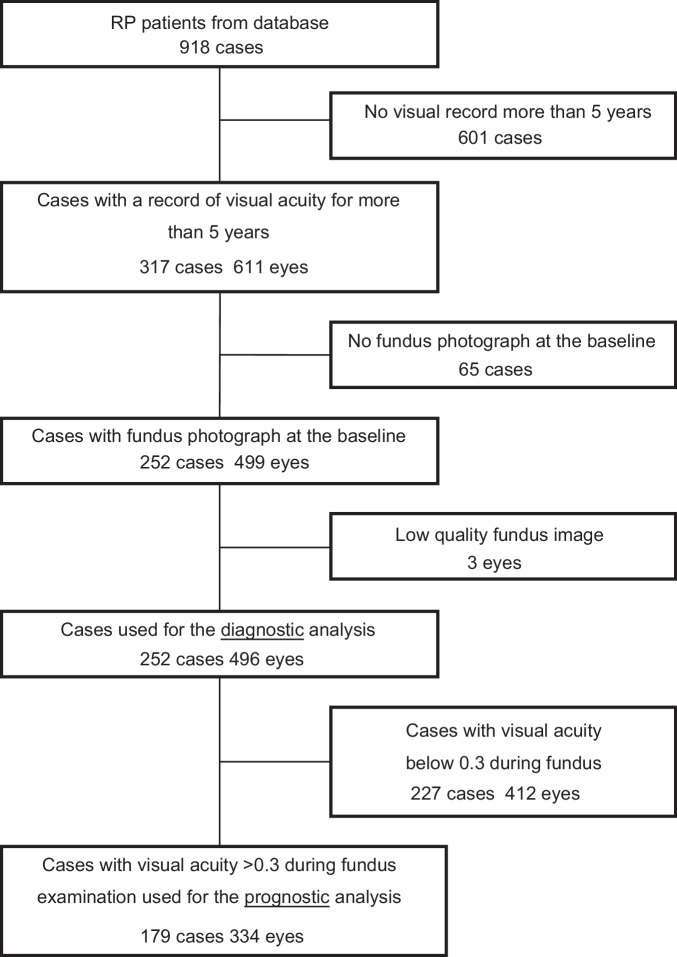
Flow diagram of patient selection. The data from 918 patients with retinitis pigmentosa (RP) were collected from the Electronic Medical Record system. After removal of cases lacking visual records for 5 years and baseline fundus photograph, additional three eyes were excluded because of unsuitable fundus image, such as blurred one due to media opacity. Four hundred and ninety-six eyes from 252 cases were used for diagnostic analysis. Lastly, cases with visual acuity below 0.3 at the time of fundus examination were used for the prognostic analysis, resulting in 334 eyes from 179 cases.

The patients with RP were extracted from the database at Chiba University Hospital Electronic Medical Record system. A total of 918 patients with RP were initially included (Fig. 1). After excluding cases without visual records for more than 5 years, cases without fundus photographs at the baseline, the cases with unsuitable fundus images, such as blurred photographs, 496 eyes in 252 cases were used for the diagnostic analysis. Cases with visual acuity below 0.3 at the time of fundus examination were excluded when examining the relationship between visual prognosis and fundus images. Eventually, 334 eyes in 179 cases were used for the prognostic analysis. The patients’ demographics at the baseline are presented in Table 1. The majority of patients are sporadic (75.4%) and only one patient (0.4%) had X-linked recessive inheritance. There are only three eyes (0.6%) with glaucoma showing mild enlargement of optic disc cupping and four eyes (0.8%) with macular degeneration.

**Table 1:** Patient demographics at baseline

Number of cases	252
Number of eyes	496
Age (mean ± SD)	54.1 ± 15.7
Sex	
Male	131 (52%)
Female	121 (48%)
Mode of transmission	
AD	27 (10.7%)
AR	34 (13.5%)
XL	1 (0.4%)
Sporadic	190 (75.4%)
Best-corrected visual acuity (logMAR, mean ± SD)	0.65 ± 0.93
Mean deviation, HFA10-2 (dB, mean ± SD)	-16.9 ± 10.7
Lens status	
No cataract	159 (32.1%)
Cataract	200 (40.3%)
IOL	133 (26.8%)
Aphakia	4 (0.8%)
Vitreous opacity	2 (0.4%)
OCT findings	
Ellipsoid zone: present	333 (67.4%)
Macular edema	39 (7.9%)
Comorbidity	
Glaucoma	3 (0.6%)
Corneal opacity	2 (0.4%)
Macular degeneration	4 (0.8%)
Vitreo-macular traction	3 (0.6%)
Epiretinal membrane	13 (2.6%)

*AD* autosomal dominant inheritance, *AR* autosomal recessive inheritance, *HFA* Humphrey field analyzer, *IOL* intraocular lens, *OCT* optical coherence tomography, *SD* standard deviation, *XL* X-linked recessive inheritance.

### Verification of RP diagnostic performance using existing models

**Fig. 2:**
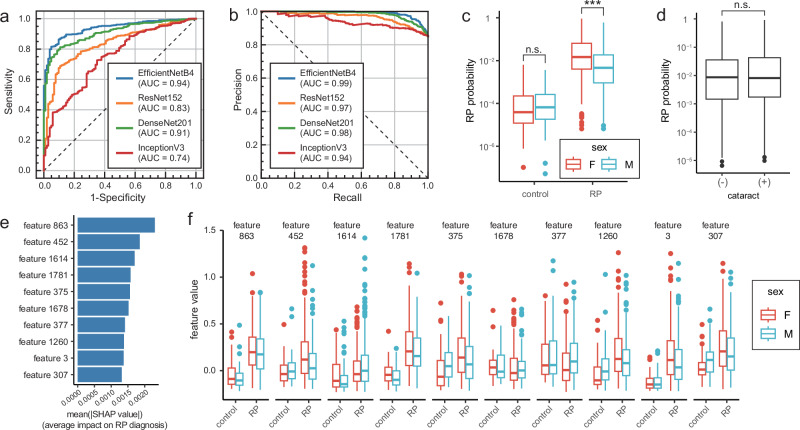
RP diagnosis using existing DL models. **a** Receiver operating characteristic (ROC) curves and their AUROC values for the different DL models in diagnosing RP. EfficientNetB4 achieved the highest AUROC of 0.94. ResNet152 and DenseNet201 also had better performance than InceptionV3, achieving AUROC of 0.83, 0.91, and 0.74, respectively. **b** Precision recall curves (PRCs) and their AUPRC values for the DL models in diagnosing RP. AUPRC of EfficientNetB4 was 0.99, which was the best among these four algorithms (AUPRC = 0.97, 0.98, and 0.94 by ResNet152, DenseNet201, and Inception V3, respectively). **c** Diagnostic probabilities of RP by sex. The predicted probability was significantly higher in female patients compared to males (P < 0.001), whereas there was no significant difference between sexes among control non-RP patients. **d** Diagnostic probabilities of RP by presence of cataract. The presence of cataracts had no significant effect on the probability of RP diagnosis. **e** SHAP scores for the top 10 diagnostic image features in RP. Feature 863 has the highest SHAP value. **f** Image feature values by sex in RP and control patients. Features 863, 452, 1781, 375, 1260, 3, and 307 showed higher feature values in the eyes with RP than those in the controls, with this trend being more pronounced in female patients.

Figure 2 shows the performance of the different DL models in diagnosing RP. Four models were tested to determine the best model for further analysis. As the AUROC was highest with EfficientNetB4 (AUROC = 0.94, Fig. 2a), we adopted this model for subsequent analyses. ResNet152 and DenseNet201 also had better performance than InceptionV3 (AUC = 0.83, 0.91, and 0.74, respectively), but these models were inferior to EfficientNetB4. Figure 2b shows the precision recall curve and the area under the precision recall curve (AUPRC) of EfficientNetB4 was 0.99, which was the best among these four algorithms (AUPRC = 0.97, 0.98, and 0.94 by ResNet152, DenseNet201, and Inception V3, respectively). The probability of RP diagnosis was significantly higher in female patients than in male patients (P < 0.001, Fig. 2c). The difference in the RP diagnostic probability between the sexes was also observed in other models, excluding InceptionV3 (Supplementary Fig. 1). However, the predicted probabilities for male and female were identical in the control group. Therefore, the high female probability is not an intrinsic tendency of this model, but it is unique in RP cases. The presence of cataracts did not affect the probability of RP diagnosis (Fig. 2d). The SHAP values were calculated for 1,792 image features obtained with the EfficientNetB4 model, and the top 10 features with the highest SHAP values are shown in Fig. 2e. Feature 863 has the highest SHAP value. Among these 10 highest SHAP features, 863, 452, 1781, 375, 1260, 3, and 307 showed higher feature values in the eyes with RP than those in the controls. The box plot also shows that the influence of gender varies among the features (Fig. 2f).

**Fig. 3:**
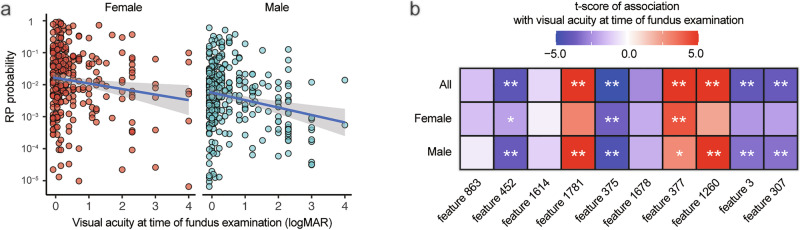
Relationship between the RP diagnostic performance and visual acuity. **a** Relationship between the probability of diagnosis of RP and visual acuity during fundus examination. The diagnosis of RP with this model is superior for the cases with good vision, while it is less effective for eyes with very low vision. **b** Relationship between key image features for RP diagnosis and visual acuity during fundus examination. The reduced diagnostic probability in low-vision patients is likely due to the attenuation of key image features critical for RP prediction. This trend is more evident in male patients.

The probability of RP diagnosis was higher in cases with higher visual acuity and lower in those with lower visual acuity (Fig. 3a). This model performs well in diagnosing RP for cases with good vision but is less effective for those with very low visual acuity. The low probability of the diagnosis in cases with very poor vision can be a result of fewer observed characteristics in the images. This trend is more evident in the male patients (Fig. 3a). SHAP values of features 1781, 377, and 1260 were positively correlated, whereas those of 452, 375, 3, and 307 were negatively correlated with visual acuity (Fig. 3b). In male patients, the contribution of more features is significantly affected by visual acuity, which partially accounts for the low diagnostic probability for males (Fig. 3b, bottom row).

**Fig. 4:**
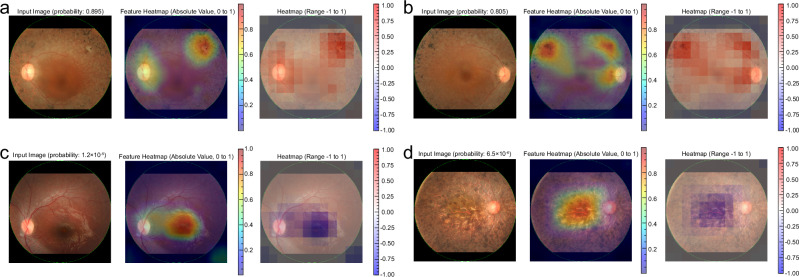
Representative cases and feature heatmaps for RP diagnosis. **a** A case with a high probability of RP diagnosis. The left eye of a 46-year-old female patient with RP had a best-corrected visual acuity of 1.0 (left). The probability of RP diagnosis was high at 0.895. The feature heatmap based on the SHAP values highlighted hot spots in the pigmented area at the superior temporal region and the optic disc (center). The feature heatmap, which considers the sign of the SHAP values, showed positive contributions in this area, suggesting that these features contribute to a higher probability of RP diagnosis (right). **b** Another case with high probability of RP diagnosis. The right eye of a 38-year-old female patient with RP had a best-corrected visual acuity of 0.7 (left). The probability of a diagnosis of RP was high at 0.805. The feature heatmap identified hot spots in the pigmented area at the superior temporal region and the region adjacent to the optic disc (center). The signed feature heatmap showed positive contributions of these features (right). **c** A case with poor probability of RP diagnosis. The left eye of an 8-year-old male patient with RP. His best-corrected visual acuity was 1.0 (left). The probability of RP diagnosis was low at 1.2 × 10^-5^, likely due to the less phenotypic change in the image. The feature heatmap showed hot spot in the macular region (center). The signed feature heatmap showed negative contributions across the whole image, suggesting that these features lower the RP diagnostic probability (right). **d** Another case with poor probability of RP diagnosis. The right eye of a 57-year-old female patient with RP had a best-corrected visual acuity of light perception (left). The probability of a diagnosis of RP was low at 6.5 × 10^-6^, likely due to the very severe retinal degeneration including macula. The feature heatmap showed a hot spot around the macula where prominent pigmentation is already observed (center). Signed feature heatmap showed negative contribution across the entire image (right).

The heatmap visualization of key diagnostic features is presented in Fig. 4. The hot spots presented in red correspond to the area which the algorithm focuses on intensively for calculating the probability. The images with high probability of RP diagnosis have hot spots at pigmented areas and optic disc (Fig. 4a, b). The pigmented area is typical in RP and the optic atrophy is also phenotypic. The low probability of RP diagnosis occurs in cases with little pigmentation at the early stage (Fig. 4c) and in cases with very dense pigmentation covering the entire image at the advanced stage (Fig. 4d).

### Predicting visual prognosis in RP based on image features

Next, we constructed a prognostic model for predicting future visual acuity loss in patients with RP using machine learning survival analysis based on image-derived features. All prognostic modeling was conducted using a strict patient-level split to prevent information leakage between eyes of the same patient.

**Fig. 5:**
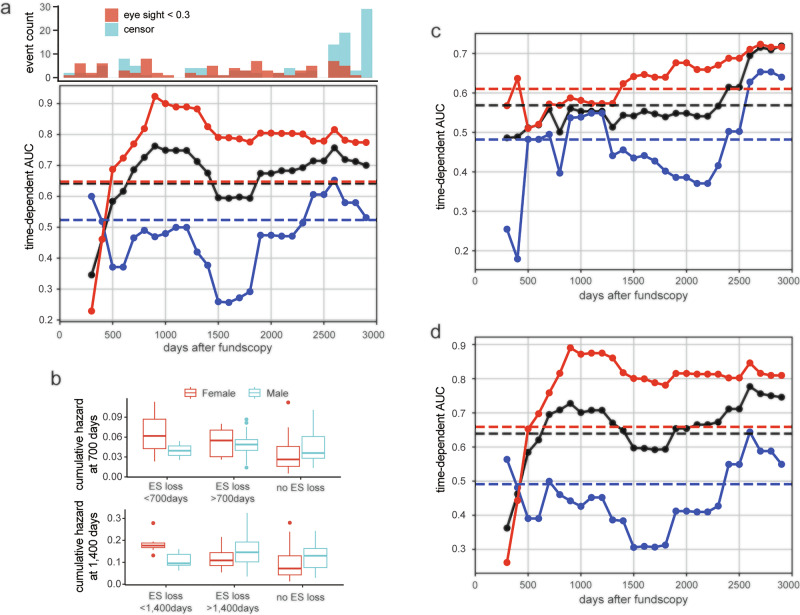
Prognostic prediction of RP using image features. **a** Frequency of events of visual acuity loss <0.3 or censoring represented by a histogram (top panel). The time-dependent AUC was high between 500 and 1400 days after the fundus examination and rapidly decreased afterward (bottom panel) and was consistently higher in female patients (red) compared to males (blue). **b** Risk of visual acuity loss as calculated by the RSF model. The 700-day cumulative hazard, representing the probability of visual acuity loss within 700 days, was calculated by sex using the RSF model (top panel). The cumulative hazard is highest in eyes that lost vision within 700 days in both sexes. The cumulative hazard at day 1400 (bottom panel) was the same for male patients who experienced visual acuity loss before and after 1400 days. **c** The time-dependent AUC for a survival analysis model based only on clinical variables including the left and right eye, age, sex, presence of cataracts, and presence of cataract surgery. The predictive performance of this model was inferior to that of the image feature-based prognostic prediction model, especially for female patients. **d** Key prognostic image features presented for each group: visual acuity loss within 700 days, visual acuity loss after 700 days, and no visual acuity loss. Features 466, 839, 1654, and 274 demonstrate the highest values in the visual acuity loss group before 700 days, while features 762, 1669, and 1390 achieve the highest values in the visual acuity loss group after 700 days. Features 432 and 1539 show elevated values in cases without visual acuity loss, whereas feature 1100 exhibits no substantial difference across groups.

Figure 5a shows the performance of the image-only model, which used DL features extracted from the EfficientNetB4 architecture. The time-dependent AUC remained high between 700 and 1400 days after fundus photography, indicating that the model is particularly effective in forecasting vision decline within this mid-term window. Notably, the performance was consistently better in female patients compared to male patients across the entire observation period.

Cumulative hazard was calculated on day 700 and on day 1400 (Fig. 5b). For female patients, the cumulative hazard at day 700 was highest in the group that experienced visual acuity loss within 700 days, moderate in the group that experienced visual acuity loss after 700 days, and lowest in the group that did not experience visual acuity loss, which was consistent with the actual visual acuity loss events. This trend for female patients was also observed in the cumulative hazard at 1400 days. However, in male patients, there was little difference in cumulative hazard between groups. This explains the low time-dependent AUC in the male patients throughout the study period.

Figure 5c shows the time-dependent AUC of a metadata-only model, which predicts visual acuity decline based solely on clinical variables, such as age, sex, laterality, presence of cataract, and history of cataract surgery. While the model demonstrated modest predictive ability at the late phase of follow-up, its overall performance was inferior to the image-based model shown in Fig. 5a, particularly among female patients. This contrast highlights the added prognostic value of fundus image features over standard clinical parameters, especially for medium-term risk estimation. The metadata-only Cox PH baseline yielded c-index of 0.52 overall and 0.56 in females, which was lower than the RSF baseline evaluated under the same split (Supplementary Fig. 2).

Figure 5d presents the time-dependent AUC of the hybrid model, which combines both image-derived features and clinical variables. Compared to the image-only (Fig. 5a) and metadata-only models (Fig. 5c), the hybrid model improved prediction performance at many time points. In particular, the model showed robust and stable AUC values between 500 and 1500 days, indicating that integrating clinical context with fundus image features improves prognostic accuracy.

High performance in visual acuity prognosis was achieved even when considering important image features individually. The predictive performance for each feature varies substantially over time (Supplementary Fig. 3). In Supplementary Fig. 4, the feature values are displayed separately for three groups: those experiencing vision loss before 700 days, those after 700 days, and those without vision loss. The trends in the feature values vary across features.

The multiple regression analyses were performed by the baseline values for the cutoff visual acuity of 0.3 during observation period. The odds ratio of retinal sensitivity measured by Humphrey Visual Field Analyzer was 0.96 (P = 0.026, CI: 0.926- 0.995).

### Differences in important image features in diagnostic and prognostic prediction

**Fig. 6:**
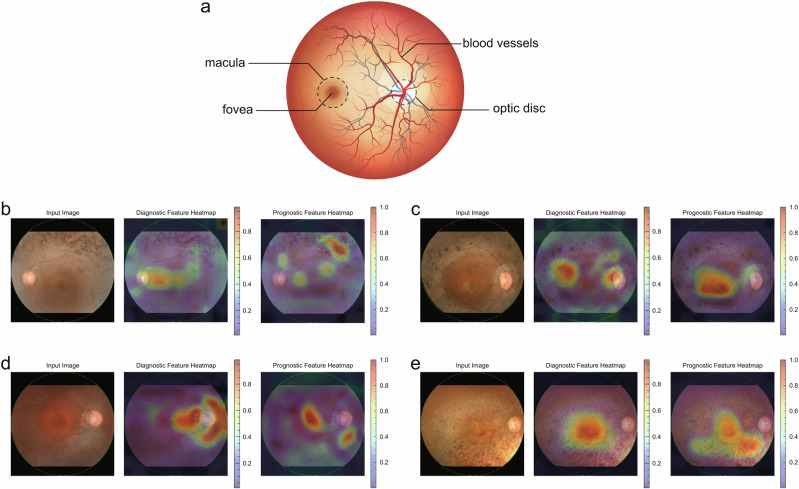
Comparison of key features for diagnosis and prognosis prediction. **a** Schematic representation of a fundus image highlighting the main structures. The fundus image from Adobe Stock was used under a Standard License, and Permission for use and adaptation has been obtained (https://stock.adobe.com/jp/search?k=fundus&search_type=usertyped&asset_id=61481940). **b** A case with visual acuity dropping below 0.3. The left eye of a 38-year-old male patient with RP had an RP diagnostic probability of 0.160 (left). The diagnostic feature heatmap showed hot spots in the pigmented area at the superior temporal, optic disc, and macula (center). The prognostic feature heatmap showed hot spots around the macula, along the superior arcade vessels, and in the pigmented area at the superior temporal region (right). His visual acuity dropped from 1.2 to <0.3 at 609 days after the fundus examination. **c** Another case with visual decline. The right eye of a 39-year-old female patient with RP had a probability of RP diagnosis of 0.298 (left). The diagnostic feature heatmap shows hot spots in several pigmented areas at the temporal retina (center). The prognostic feature heatmap showed a hot spot around the macula (right). Her visual acuity declined from 0.8 to <0.3 at 407 days after the fundus examination. **d** A case with good vision throughout the studied period. Right eye of a 50-year-old female patient with RP. Her best-corrected visual acuity was 1.0 and the probability of a diagnosis of RP was 0.164 (left panel). The diagnostic feature heatmap shows hot spots in the area nasally adjacent to the optic disc, and the temporal area to the macula (center). The prognostic feature heatmap showed an intense hot spot at the macula and the area inferior to the disc (right). **e** A case with poor visual acuity throughout the period. This case was excluded from the time-dependent AUC calculation, but feature heatmaps were generated to investigate the characteristics of existing visual impairment. Right eye of a 73-year-old male patient with RP (left). The probability of a diagnosis was 0.011. The diagnostic feature heatmap showed hot spots in the macula (center). The prognostic feature heatmap showed a hot spot at the inferior temporal to optic disc and macula (right). His visual acuity was lower than 0.3 throughout the study period.

The key features for diagnosing RP are completely different from those for predicting visual prognosis. The relationships between feature importance and the top 10 features for each task are illustrated in Supplementary Fig. 5. Interestingly, there is no overlap in top important features between tasks, suggesting that the diagnostic features do not sufficiently predict the prognosis, and vice versa. The differences in key features between the diagnostic and prognostic models are illustrated as heatmaps in Fig. 6c–f, and Fig. 6b shows a schematic diagram of the fundus image and the main structures. The cases with visual declining crossing 0.3 in decimal units had hot spots at the macula and surrounding area (Fig. 6c, d). The patterns of the heatmap differ from those for diagnosis which mainly focus on the pigmented area. The cases maintained good visual acuity (>0.3) showed few hot spots in the macula (Fig. 6e). Similarly, the cases with poor visual acuity (<0.3) throughout the study period had fewer hot spots around the macular area (Fig. 6f).

## Discussion

In this study, we have developed a prognostic prediction model of visual acuity in the eyes with RP by fine-tuning an existing diagnostic model. First, we confirmed the performance of four DL models in diagnosing RP. EfficientNetB4 had the best performance, and this was used for further analysis. EfficientNet is known to achieve high performance with a relatively small number of parameters by carefully balancing the network’s depth, width, and resolution, and is also highly effective for transfer learning^17^. The high diagnostic performance of RP in this study also demonstrates the generalizability of EfficientNet to populations different from the training data. Model performance was associated with visual acuity during fundus examination, with cases of higher visual acuity being more accurately detected. The model performed well because the cases with good visual acuity have normally appeared macula and were correctly evaluated by the model. Eventually, we developed the prognostic model of the eyes which predicts a visual acuity loss crossing 0.3. The prognosis prediction model demonstrated high performance, achieving an average time-dependent AUC of 0.82 and reaching 0.87 specifically for female patients. Although there have been several DL models to diagnose RP, this is the first report of a model predicting the prognosis of longitudinal visual function in RP-affected eyes.

The diagnostic performance was the best if EfficientNetB4 was used, achieving an AUROC of 0.94 and an AUPRC of 0.99. The diagnostic performance of this model based on the color fundus photography was comparable to the previous models. Li et al. have reported an AUROC of 0.99 for a model using the SeResNext50 algorithm, using 137 color fundus images for validation and 587 images for training^18^. In this study, we used 496 eyes to validate the existing model trained on 1920 fundus images, achieving favorable outcomes. This can be attributed partly to the consistently high quality of the fundus images used and the precise cropping of all images. The heatmap visualization of key diagnostic predictors showed the areas concerned at the pigmented area, macula, and optic disc in most cases. On the other hand, the optic disc appeared hot especially in the dark and obscure images. The change around the optic disc is difficult to perceive and is hardly noticed even by experienced ophthalmologists. This shows the potential of the DL model to detect very early changes in the fundus image and delineate the RP cases before pigmentary change is observed in the area captured by 50-degree fundus camera (Supplementary Fig. 6). The treatment strategy for RP has just been emerging. The recent advancement of gene therapy including Luxturna^R^ (targeting *RPE65*) shows promising results in a small part of RP patients who have a wide variety of gene mutations^2^. The effect of those therapies should be enhanced if they are used at the desirable timing which is usually an early stage in the disease course. The timely diagnosis of RP may contribute to drawing the desired response of the treatments. In addition, if the AI model can predict the disease course, it can be used as a screening for cases who require treatment to maintain their vision. For those who seem to lose vision in the near future, the treatment should be given faster than those who do not. At the same time, the risk of overtreatment and adverse events should be considered because the capability of prognostic decision is not always perfect.

We developed the prediction model of the visual acuity in the eyes with RP. So far, there has not been reported longitudinal visual acuity in RP eyes using DL models. For patients with age-related macular degeneration (AMD), the AI model that predicts visual acuity after anti-VEGF treatment has been developed based on optical coherence tomography images^19^. The sensitivity of the AI model was 0.615, outperforming the ophthalmologist, whose sensitivity was 0.385. Although this AMD model is useful, it lacks generalizability to other diseases. In this study, we successfully demonstrated the leverage of existing diagnostic models for highly accurate prognosis prediction. RP is a progressive disease, and no established remedy is available right now. Therefore, predicting a patient’s visual prognosis is critical. The visual function in daily activities declines faster in some patients than is estimated by the ophthalmological tests^20^. Low vision care is more effective when initiated in the early stage of RP, while patients still have minimally impaired vision^21^. Early initiation of low vision care also promotes more efficient use of social resources. The information provided by this study on visual prognosis provides valuable guidance for future RP management.

One of the issues often discussed regarding DL models is the “black box” nature of their inference process. In advanced RP cases, retinal findings, such as pigmentation are typically observed, and heatmap visualization identified hot spots in the pigmented regions of the retina in this study. The visualized reasoning basis of the AI model, which is consistent with the current clinical findings, provides credit that the model correctly captures the RP characteristics. On the other hand, the heatmap visualization for visual prognostic prediction shows the hot area at the macula and its surroundings. Visual acuity heavily depends on the status of the macula, and the neighboring area is also important to speculate the progression of macular degeneration. As the clinical ophthalmologist examines the patients by focusing on different parts of fundus according to what they want to know, the features associated with diagnosis and predicting visual acuity were different as shown in Fig. 6a. Predicting visual acuity solely from images is challenging even for skilled ophthalmologists, and the key image features identified by the prognosis prediction model offer novel insights into RP pathology related to visual acuity.

The model in this study showed higher predicted probability in the female patients than in the male patients, both for diagnosis and prognosis. The reasons for this better performance in female cases are not fully elucidated, but some speculations would be made as follows: 1) The EfficientNetB4 model used in this study prominently detected features in the fundus image of female than those of the male. The specific feature contributing to the high performance in female patients is difficult to determine, but the combination of features may contribute to the performance. 2) In general, the probability of the fundus images by AI models tends to be higher among women than men. The gender can be determined using AI algorithm with high accuracy, such as AUC of 0.94–0.97^22,23^. In such case, the probability is higher in the female subjects. Gerrits et al. investigated the effect of gender and age on the prediction of cardiovascular risk based on the fundus images and the prosperity of probability was observed in the female cases^24^. The model can identify the gender with high AUC 0.97 and this innate gender recognition may affect the probability of the other parameters. However, they did not explain the exact reason for this woman’s tendency. 3) The original AI model adopted in this study might have been trained by biased data. The performance of model is influenced by the data used during training and it can be stronger in assessing the female images if most of the training data was derived from female cases. Unfortunately, the RFMiD dataset used to train the original AI model does not include background information, such as gender or age. Other possibility is genotypic difference between men and women. We did not have the information on genotype, but there was no significant difference in the mode of inheritance between male and female patients (P = 0.554, Chi-square test).

The quality of visual prognostic prediction was further improved by combining with the information on baseline visual acuity (data not shown). Previous studies have reported that the accuracy of RP diagnosis using DL models is improved by combining OCT (optical coherence tomography) images with color fundus images^25^. The multimodal imaging consists of fundus images, OCT, and other image modalities and functional assessments (Table 2) are vital for more precise prediction and need to be further investigated. Unfortunately, the genotypes of patients were not available in this study, the other AI model based on FAF, IR and OCT can predict the causative gene^26^. The information of genotypes may enhance the accuracy of the diagnosis of RP. Moreover, the genotype is related to disease prognosis and contributes to the prediction of visual course. Other approaches including genotypes have also been reported and the longitudinal analysis of decreasing FAF area was possible using a novel AI model^27^.

**Table 2:** Studies of the relationship between visual function and images of RP using AI models

Authors	Year	Subjects (patients)	Purpose	Modality	Models	Performance
Liu et al.^25^.	2023	RP (314)	Determine BCVA < 20/40	SLO-IR, OCT	AlexNet, DenseNet-161, ResNet-50, ResNet-152	AUROC = 0.85 with combining SLO and OCT
Nagasato et al.^41^.	2023	RP (695)	Retinal sensitivity, BCVA	UW-pseudocolor image, UWFAF	VGG-16, ResidualNetwork-50, InceptionV3, DenseNet121, EfficientNetB0	SRC = 0.684 (mean deviation), 0.309 (BCVA)
Sumaroka et al.^42^.	2019	RP (20), LCA (18)	Predict VA after treatment	OCT	Random Forest models	Severe LCA improvement after treatment localizes to the foveal area
Yassin et al.^43^.	2023	RP (32)	Align retinal sensitivity by microperimetry and SLO-OCT	IR-microperimetry, SLO-OCT, SLO-IR	Details not given	Dice coefficient AUROC = 0.991
**This study**	**2025**	**RP (179)**	**Predict declining VA crossing 0.3**	**Color Fundus Image**	**EfficientNetB4**	**Average time-dependent AUC** = **0.82**

*AUROC* area under the receiver operating characteristic curve, *BCVA* best-corrected visual acuity, *FAF* fundus autofluorescence, *IR* infrared retinal image, *LCA* Leber congenital amaurosis, *RP* retinitis pigmentosa, *OCT* optical coherence tomography, *SRC* standardized regression coefficient, *SLO* Scanning laser ophthalmoscopy, *UW* ultrawide.

While our models demonstrate strong performance in predicting visual acuity decline within this single-center dataset, several factors limit generalizability to broader RP populations. First, this study relies on retrospective data collected at a single tertiary referral center, with a patient population that may not represent broader demographic or genetic diversity—particularly in terms of ethnicity, disease subtype, or imaging protocols. Additionally, we used a single imaging device type, and calibration differences across cameras or centers could affect the portability of DL-derived features. Second, the current study lacks external validation using an independent cohort. While we took steps to obtain robust, leakage-free estimates through patient-level split and K-fold cross-validation within our dataset, external data—from other institutions, geographic regions, or screening programs—are needed to confirm real-world performance. Future prospective, multi-center studies are needed to validate both the image-only and hybrid models across diverse clinical settings. Third, even within our dataset, model performance varied by sex and follow-up period, as shown in Fig. 5. While the hybrid model improved overall performance, AUC values dropped in male patients during long-term follow-up. We hypothesize this may reflect sex-related differences in disease progression or differences in clinical management, but further validation is required. Fourth, the existing model we used was based on a relatively outdated architecture, and the fundus image dataset used for training was not up-to-date. In recent years, emerging architectures like the Vision Transformer have outperformed conventional CNNs in various image diagnosis tasks^28^. New datasets of fundus images are being constructed^29,30^, aiming to include a greater variety of eye diseases and more images. Utilizing these datasets containing more RP cases, combined with the latest DL architectures for pre-training, enables the capture of additional RP features and enhances the performance of both diagnosis and prognostic prediction. Fifth, the follow-up data on visual acuity is limited to an average of five years, and long-term information over ten years or more is lacking. The model predicted visual decline precisely in a 700-day and 1400-day period for female patients, which may not be sufficient for a slowly progressive condition. RP is a slowly progressive disease, and a longer period of observation is necessary for detailed observation. With an average observation period of 5 years, it is reasonable that the prediction model demonstrated strong prognostic performance, particularly in the first 1500 days. Taken together, we emphasize that while our findings are promising, they should be interpreted with caution outside the context of this dataset and imaging protocol. Future work should focus on validating these models using multi-institutional external cohorts and prospectively collected data from diverse populations and imaging devices. It will also be essential to assess the fairness of the models across demographic and phenotypic subgroups and to evaluate their clinical utility through integration into real-world clinical workflows, ideally with physician-in-the-loop feedback and oversight. By openly acknowledging these limitations and identifying concrete directions for future validation, we believe our study offers a transparent and practical foundation for clinical translation, while maintaining scientific rigor and responsibility.

In conclusion, we have reported the diagnosis and prediction model for visual prognosis in patients with RP by fine-tuning the existing AI model for retinal images. The performance of the model was outstanding and achieved high diagnostic accuracy for RP. The model also had a superior performance in predicting the visual prognosis and can bring benefits for the patients to plan on utilizing social support to maintain their daily lives. In order to make the model useful for the community, the way to implement it in daily practice needs to be deliberated.

## Methods

### Study Patients

A total of 918 patients with RP from an EMR-based database at Chiba University Hospital were reviewed. All cases included have Asian ethnicity. The diagnosis of RP was confirmed by at least two board-certified ophthalmologists. The cases without a record of visual acuity for five consecutive years were excluded from this study. Sixty-five cases lacked the fundus photograph at the baseline when the first measurement of visual acuity was performed. Among the cases with the baseline fundus photograph, three eyes were excluded because of the unsuitable fundus images. Those images had a poor resolution because of dense cataracts and poor mydriasis in one eye each, and no abnormal finding in the fundus image in one eye with retinal degeneration in the far peripheral retina. Finally, 496 eyes in 252 RP cases were used for diagnostic analysis. We used 103 eyes from patients with non-RP eye diseases as control samples for RP diagnosis. The visual prognosis was predicted for 334 eyes (179 cases) whose visual acuity was greater than 0.3 at the baseline. The dataset was divided differently for the diagnostic and prognostic analyses. For the RP diagnosis model, we used pre-trained DL models to extract features from all available fundus photographs without applying any training-test split, since the classification probabilities were derived in a feature-extraction mode without model re-training. In contrast, for the visual prognosis prediction, we performed a strict patient-level split to avoid any potential information leakage between the eyes of the same patient. Specifically, the 334 eyes from 179 RP patients with baseline visual acuity greater than 0.3 were split into training and test datasets in a 2:1 ratio, ensuring that both eyes of the same patient were assigned to only one of the datasets. All subsequent survival analyses and model evaluations—including image-only, clinical-only, and hybrid models—were conducted using this leakage-free partitioning. We note that in a prior analysis (resubmission cycle), a subset of prognostic experiments, including those based solely on clinical variables, had inadvertently used an eye-level split, which can inflate out-of-sample performance due to bilateral symmetry in RP. All results reported in the present revision are based on the patient-level split.

### Compliance with ethical guidelines

The study protocol was approved by the Institutional Review Board of Chiba University Hospital (No. HK202401-13), and the procedures conformed to the tenets of the Declaration of Helsinki. The requirement for individual written informed consent from the patients was waived by the Institutional Review Board owing to the use of retrospective and de-identified data.

### Clinical data

Clinical information at the time of fundus photography, including age, sex, visual acuity, complications, and cataract status was collected from electronic medical records. In addition, we extracted the prognosis as visual acuity loss (1 for loss, 0 for no loss) and time to event. Time to event, expressed in days, was defined as the time from fundus photography to visual acuity loss for the visual acuity loss group, or to the end of observation for the no visual acuity loss group.

### Visual acuity

For statistical analysis, the visual acuity was converted to logMAR (logarithm of minimum angle of resolution) units. The formula for calculating logMAR visual acuity is logMAR = -log(V). V: Visual acuity. The extremely low visual acuity, no light perception was graded as 4.0 logMAR units, light perception = 3.0 logMAR units, hand motion = 2.3 logMAR units, counting fingers = 2.0 logMAR units as reported by Schulze-Bonsel et al.^31^.

### DL models

In this study, we repurposed existing large-scale diagnostic models^9^ based on four different DL architectures: ResNet152, InceptionV3, DenseNet201 and EfficientNetB4^32–35^. The original models and the information on data preprocessing, augmentation strategies, and the hyperparameters used during the training process can be obtained at https://github.com/frankkramer-lab/riadd.aucmedi. The models were trained using 1920 fundus images from the Retinal Fundus Multi-Disease Image Dataset (RFMiD)^36^. Internal validation demonstrated its ability to differentiate between 46 diseases with a mean AUROC of 0.99, including RP. Since no training was performed on our RP dataset for diagnosis, data splitting was not required for this stage. In the visual acuity prognostic prediction, we applied the EfficientNetB4 model in a feature extraction mode to obtain image representations for downstream prognostic modeling. Specifically, for each fundus photograph, we extracted the output of the avg_pool layer from the EfficientNetB4 model, yielding a 1792-dimensional feature vector per image.

### Metadata-only baselines

For metadata-only baselines, we evaluated two complementary survival models:

1. Cox proportional hazards (Cox PH) with L2 regularization, and
2. Random Survival Forest (RSF) (scikit-survival), configured as in the main text.

Cox PH provides a strong, low-variance baseline when the number of clinical variables is small and well characterized; RSF can capture potential non-linearities and interactions. Both models were trained and evaluated under the patient-level split.

### Preprocessing of fundus images

The fundus images used included four types of resolution: 1200 × 872, 3696 × 2448, 2000 × 1312, and 1360 × 1024 pixels. Due to equipment limitations, the top and bottom portions of the image were missing, so the circular fundus area was extracted using the circle detection functionality of the Python cv2 package. The image was square cropped and resized to the model input size according to the neural network architecture: 380 × 380 for EfficientNetB4, 299 × 299 for InceptionV3, and 244 × 244 for other architectures.

### Random survival forests

Random Survival Forests (RSF)^37^ is a nonlinear survival analysis method based on the original random forest algorithm, capable of estimating the risk of an event occurring for each patient. In this algorithm, multiple decision trees are generated using bootstrap samples randomly extracted from the original data set, and the nodes of each tree are split based on randomly selected variables. RSF incorporates survival time information into the node splitting rule and selects variables and their thresholds at each node so as to maximize the difference in survival time between each node after splitting. Finally, the hazard function for each patient is estimated as the average of the hazard functions from all the trees. We used the RSF model implemented in the Python package scikit-survival^38^ with the default settings, except for the following parameters: n_estimators=2,000, min_samples_split=10, min_samples_leaf=15.

### Evaluation metrics

We report time-dependent AUC curves for interpretability across follow-up windows and Harrell’s c-index as a model-agnostic summary statistic under right censoring. All metrics are computed in the held-out test sets under the patient-level split.

### Evaluation of feature contribution to prediction

To evaluate feature importance in diagnosis, we used Shapley Additive Explanations (SHAP) values^39^. SHAP is a method rooted in cooperative game theory that quantifies the individual contributions of feature values to model predictions. The feature importance of the RSF prognostic model was calculated for the test data using SurvSHAP(t)^40^. SurvSHAP(t) provides a time-dependent contribution of feature values. To investigate the impact of key features on the original image, features weighted by the absolute SHAP and survSHAP(t) values were visualized as a heatmap and overlaid on the original image.

## Supplementary information

Supplementary Information

## Data Availability

The datasets generated and analyzed during the current study are not publicly available to protect individual information, including retinal vascular patterns, but they are available from the corresponding author on reasonable request.

## Code availability

The code used for model modification and data analysis in this study is made publicly available at https://github.com/eiryo-kawakami/RP_AI_paper2024. In our experiments, we used Python 3.10, and the following open-source libraries: scikit-survival = 0.22.2, pandas = 2.2.2, numpy = 1.26.4, scikit-learn = 1.3.2, survshap = 0.4.2, matplotlib = 3.8.4, tensorflow = 2.12.0, and aucmedi = 0.1.0. In visualizing the results, we used R 4.4.1, and the following open-source libraries: ggplot2 = 3.5.1, readr = 2.1.5, dplyr = 1.1.4, ggsci = 3.2.0, stringr = 1.5.1, tidyr = 1.3.1, RColorBrewer = 1.1.3, and ggrepel = 0.9.6.
