# Supplementary material for: Leveraging large scale deep learning models for diagnosis and visual outcome prediction in retinitis pigmentosa
Source: NPJ Digit Med. 2026 Jan 8;9:137. doi: 10.1038/s41746-025-02311-9 (PMC12887015; doi:10.1038/s41746-025-02311-9)
Supplement: Supplementary file 1 — Supplementary Information [file 41746_2025_2311_MOESM1_ESM.pdf]

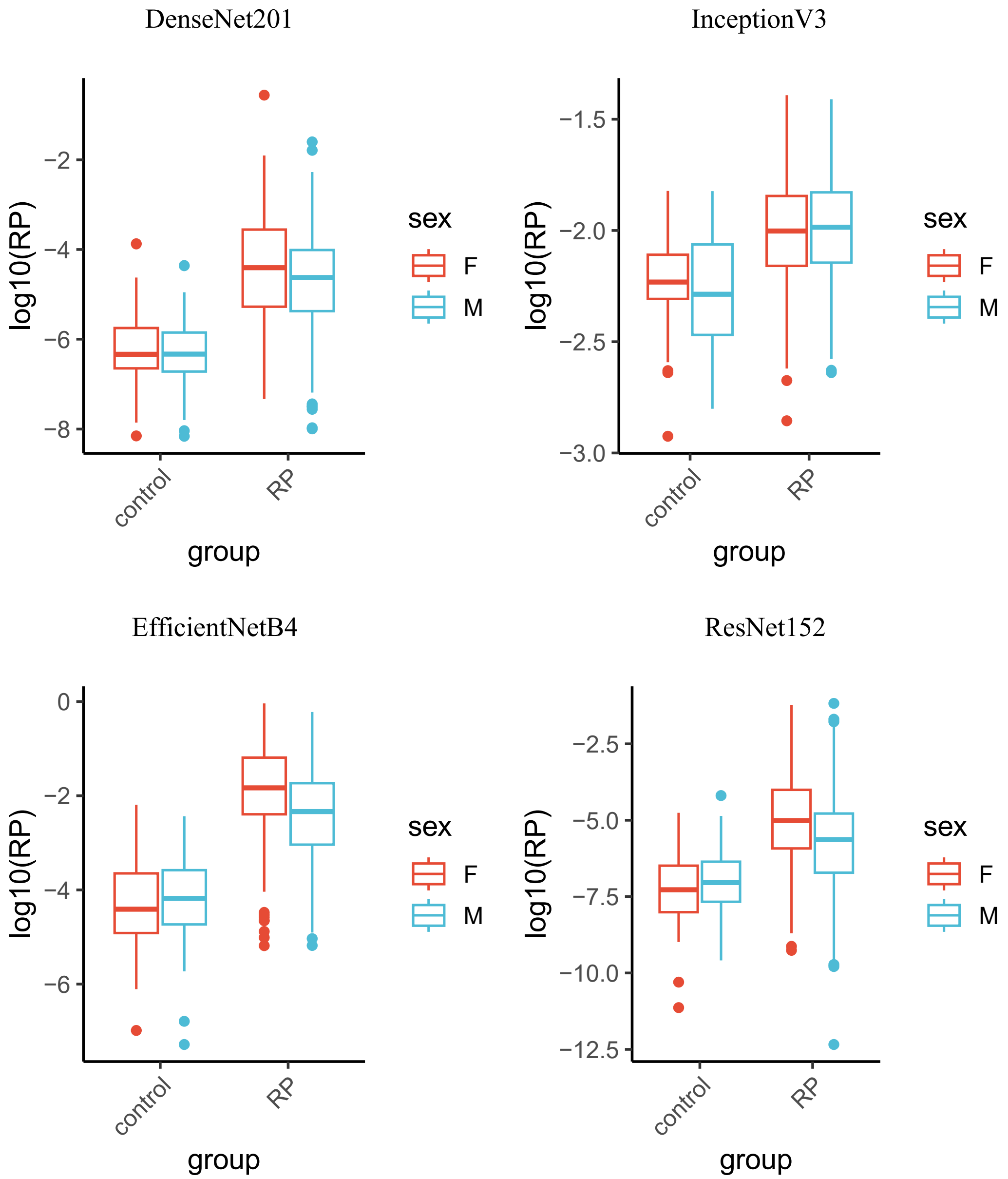

Supplementary Fig. 1: Diagnostic probabilities of RP by sex in four diagnostic models. In models excluding InceptionV3, the diagnostic probability for RP cases was higher for females.

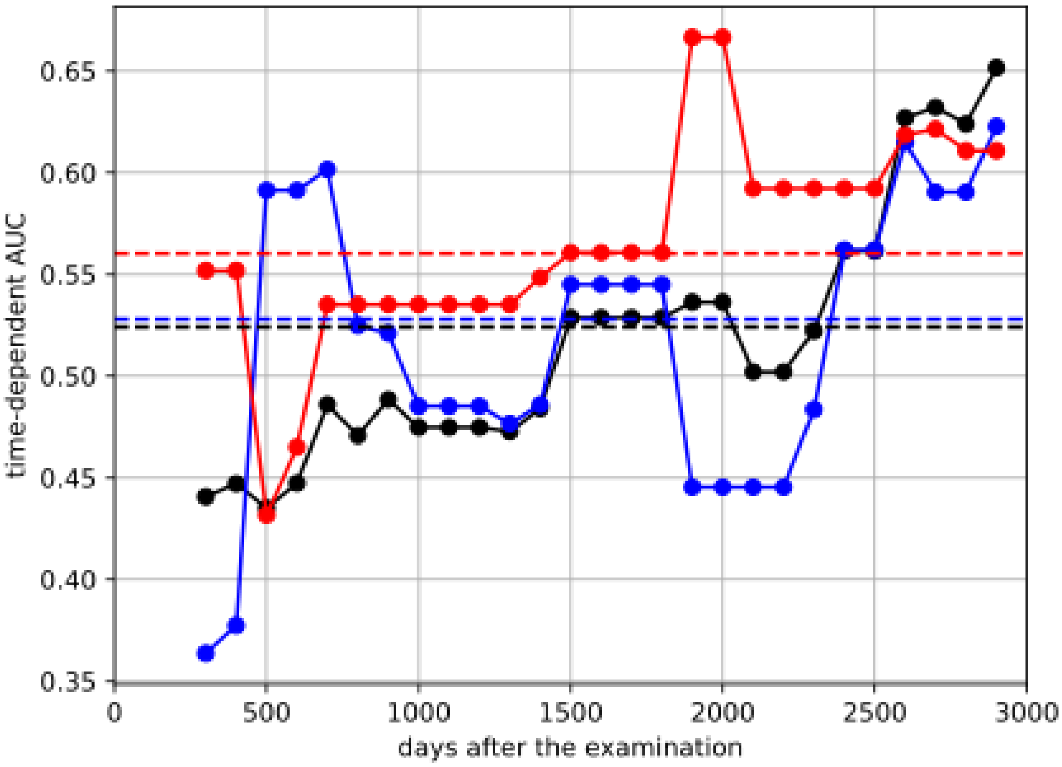

Supplementary Fig. 2: The time-dependent AUC for a metadata-only Cox PH model with L2 regularization. The predictive performance of this model was inferior to that of the image feature-based prognostic prediction model, and to that of the RSF-based metadata-only model.

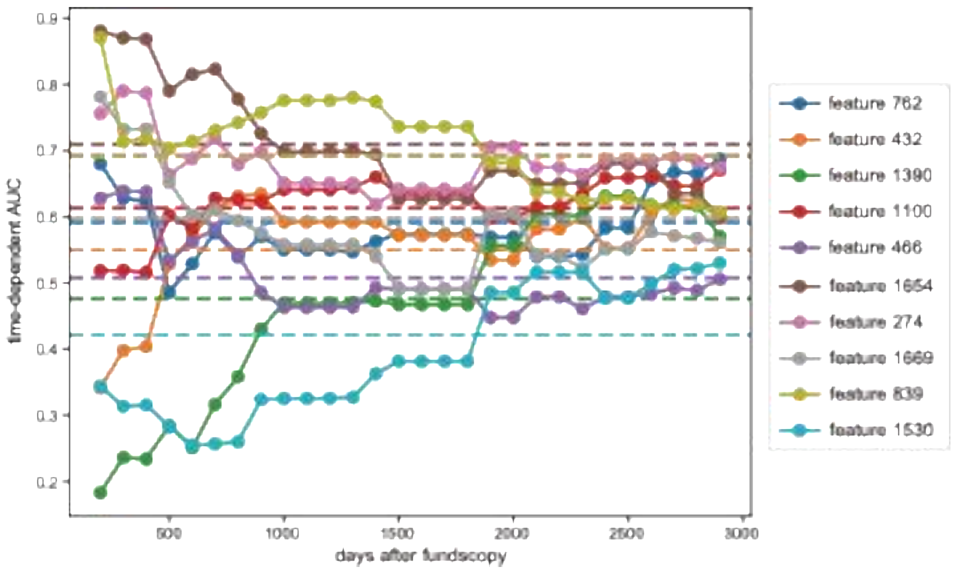

Supplementary Fig. 3: Time-dependent AUC when using individual image features to predict RP visual prognosis. Some features, such as features 432 and 1390, showed low time-dependent AUC initially but gradually increased their prediction performance by day 500, while others exhibited high predictive performance early on but declined and stabilized in the later phase.

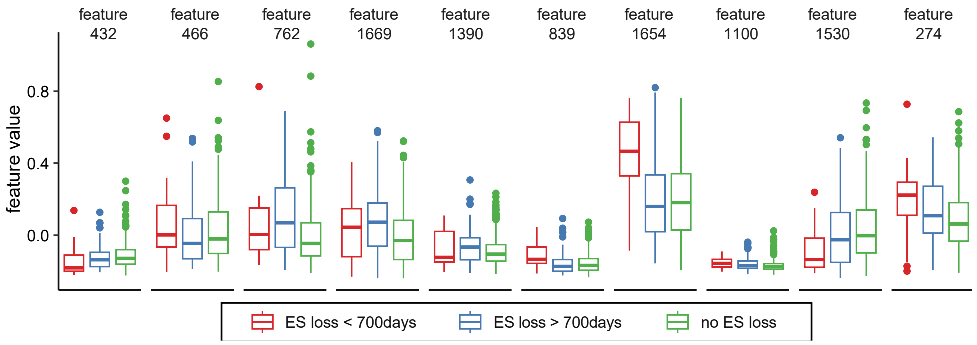

Supplementary Fig. 4: Key prognostic image features presented for each group: visual acuity loss within 700 days, visual acuity loss after 700 days, and no visual acuity loss. Features 466, 839, 1654, and 274 demonstrate the highest values in the visual acuity loss group before 700 days, while features 762, 1669, and 1390 achieve the highest values in the visual acuity loss group after 700 days. Features 432 and 1539 show elevated values in cases without visual acuity loss, whereas feature 1100 exhibits no substantial difference across groups.

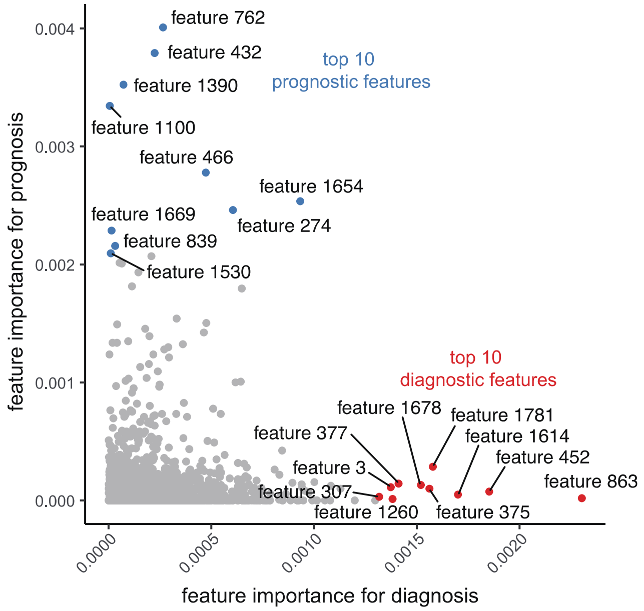

Supplementary Fig. 5: Comparison of feature importance for diagnostic and prognostic prediction.

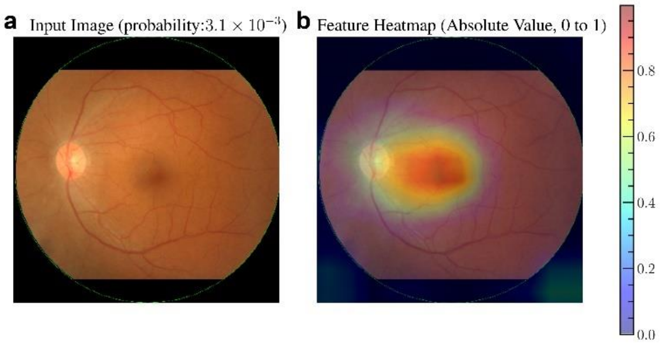

Supplementary Fig. 6: A case with a moderate probability of RP showing little pigmentary change.

a Fundus image of 65-year-old man with visual acuity of 1.0. There was little pigmentary change in the area of the 50-degree fundus photograph.

b Heat map showing a hotspot around the optic disc and macula. The probability of diagnosis of RP is 3.1×10^{-3}, which is a moderate value and shows a possibility of detecting early changes of RP before presenting pigmented degeneration at the posterior pole.
